# Supplementary material for: Integrating taxonomic signals from MAGs and contigs improves read annotation and taxonomic profiling of metagenomes
Source: Nat Commun. 2024 Apr 20;15:3373. doi: 10.1038/s41467-024-47155-1 (PMC11032395; doi:10.1038/s41467-024-47155-1)
Supplement: Supplementary file 1 — Supplementary Information [file 41467_2024_47155_MOESM1_ESM.pdf]

Supplementary Information

Supplementary Figures

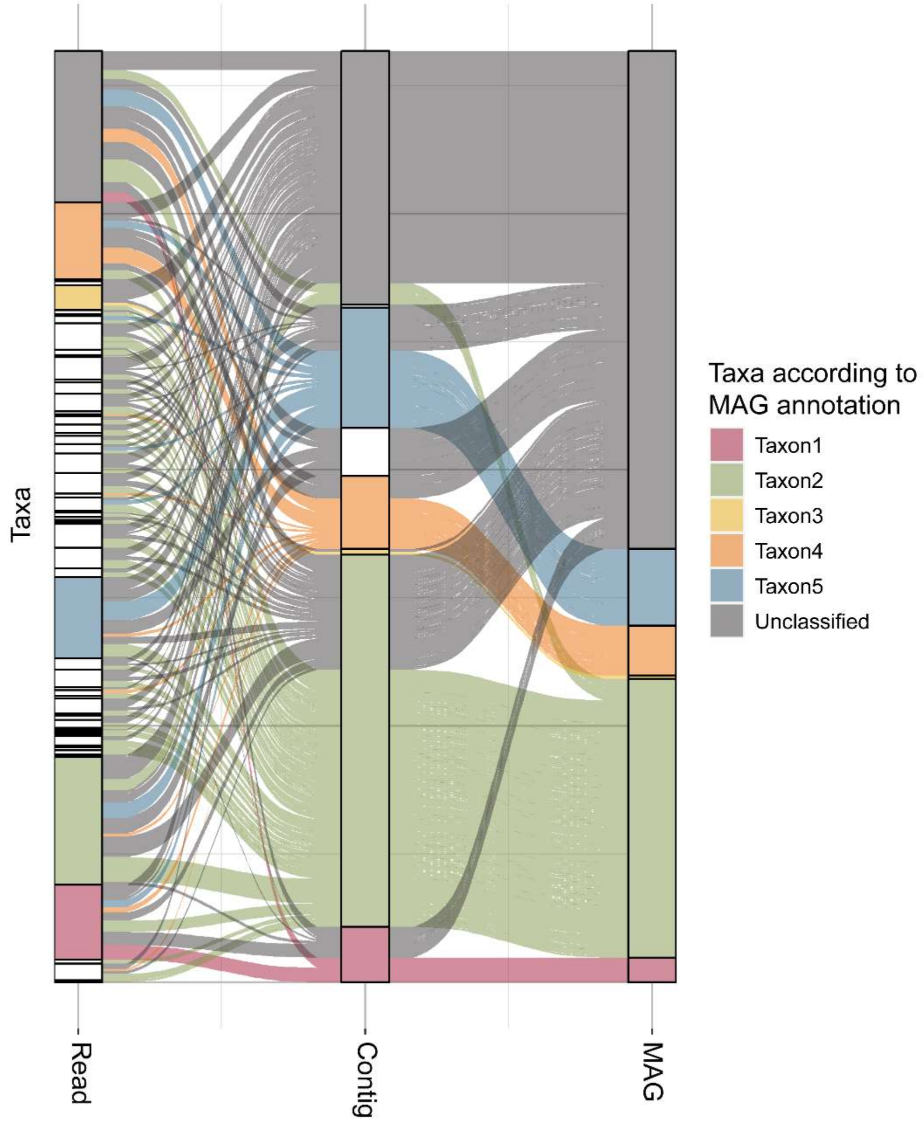

Supplementary Fig. 1 | Schematic depiction of noise reduction by using reliable taxonomic signals. Each column segment represents a taxon, each column represents an annotation step. In the read annotation step, many taxa are detected, the profile is noisy. At contig level, the number of detected taxa is much lower. Reads that were previously unannotated or had a spurious annotation now get annotated to one of eight main taxa. However, many reads that had an annotation on read level do not get an annotation on contig level, either because they don't map to any contigs, or because they map to a contig without annotation. At MAG level, there are even fewer detected taxa, and more unclassified data. Source data are provided as a Source Data file.

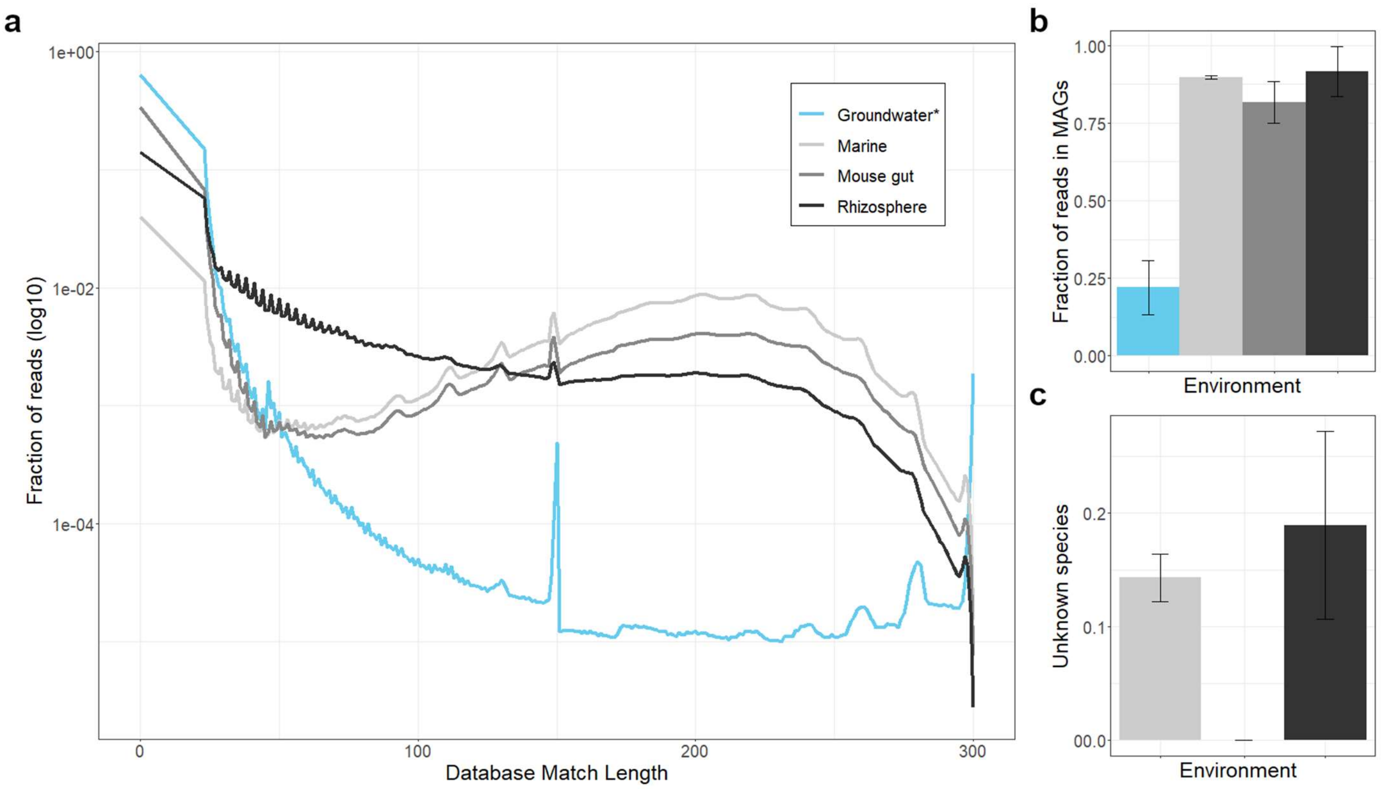

* biological dataset

Supplementary Fig. 2 | Strength and weaknesses of the different CAMI2 datasets (mouse gut: 10 samples, marine: 10 samples, rhizosphere: 8 samples, groundwater: 18 samples). a. Sequence similarity of three different simulated datasets to the database, i.e., the fraction of reads that have an exact match of the length displayed on the x axis to a sequence in nt. The biological groundwater dataset is also displayed for reference. b. Fraction of reads mapping to MAGs in the CAMI2 datasets and the groundwater dataset for reference (error bars=standard deviation). c. Fraction of reads in each simulated dataset that belong to an organism without a species representative in nt (error bars=standard deviation). Source data are provided as a Source Data file.

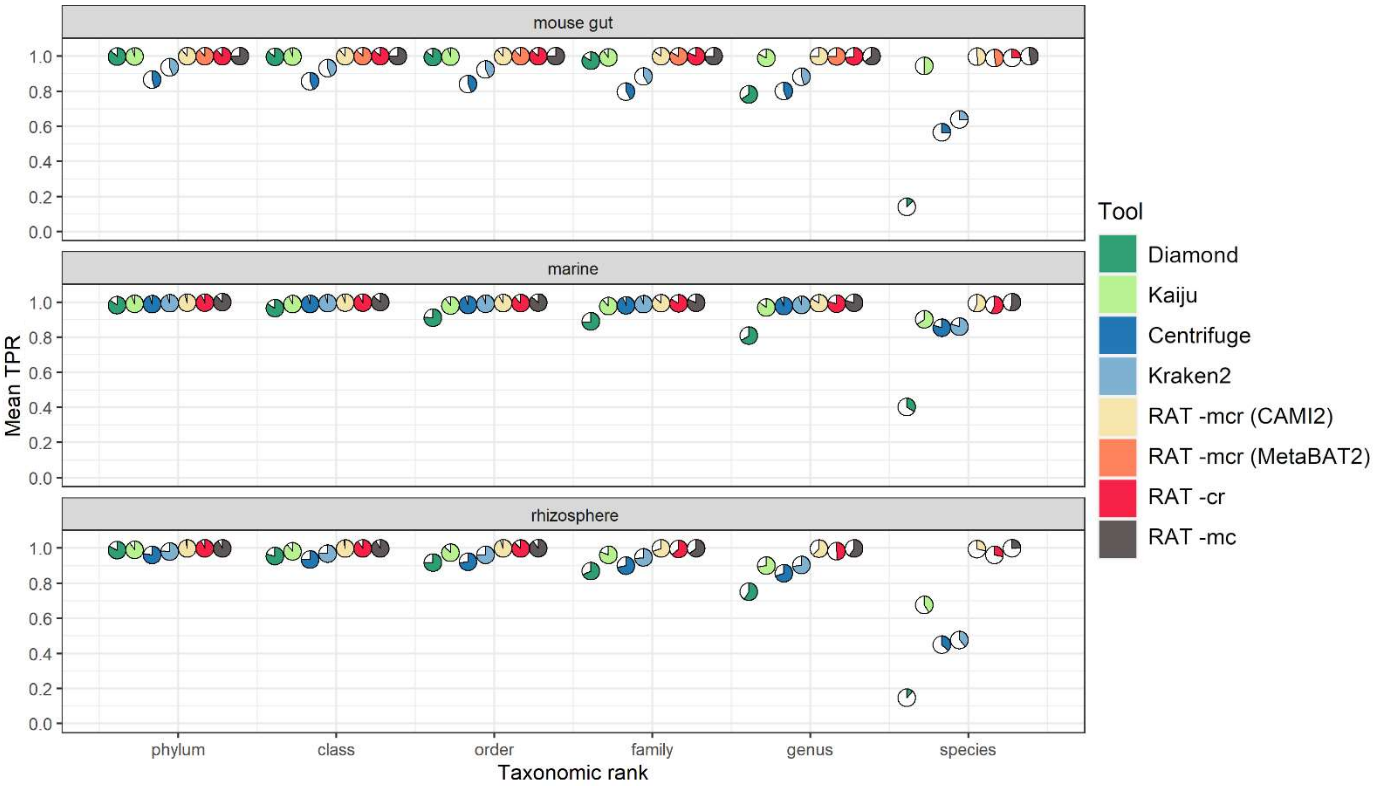

Supplementary Fig. 3. | Outcome of incorporating different taxonomic signals into read annotations on 28 samples of three challenge datasets in the CAMI2 challenge (mousegut: 10, marine: 10, rhizosphere: 8). 'DIAMOND' refers to using only direct read annotation in default sensitivity mode. 'RAT CAMI genomes' refers to a RAT -mcr run using the genomes that were provided by the CAMI2 challenge as MAG input. 'RAT MetaBAT 2 MAGs' refers to a RAT -mcr run with contigs binned by MetaBAT 2. 'RAT without MAGs' refers to a RAT -mcr run without MAG input. 'RAT -mc' refers to a RAT -mc run, using only read annotation via mapping to MetaBAT 2 MAGs and contigs, but no direct read annotation. The mean TPR refers to the fraction of correctly annotated reads per fraction of reads with any annotation averaged across the samples in the same dataset. The white section of the pie charts shows the fraction of unannotated reads per RAT run per rank. TPR, true positive rate. Source data are provided as a Source Data file.

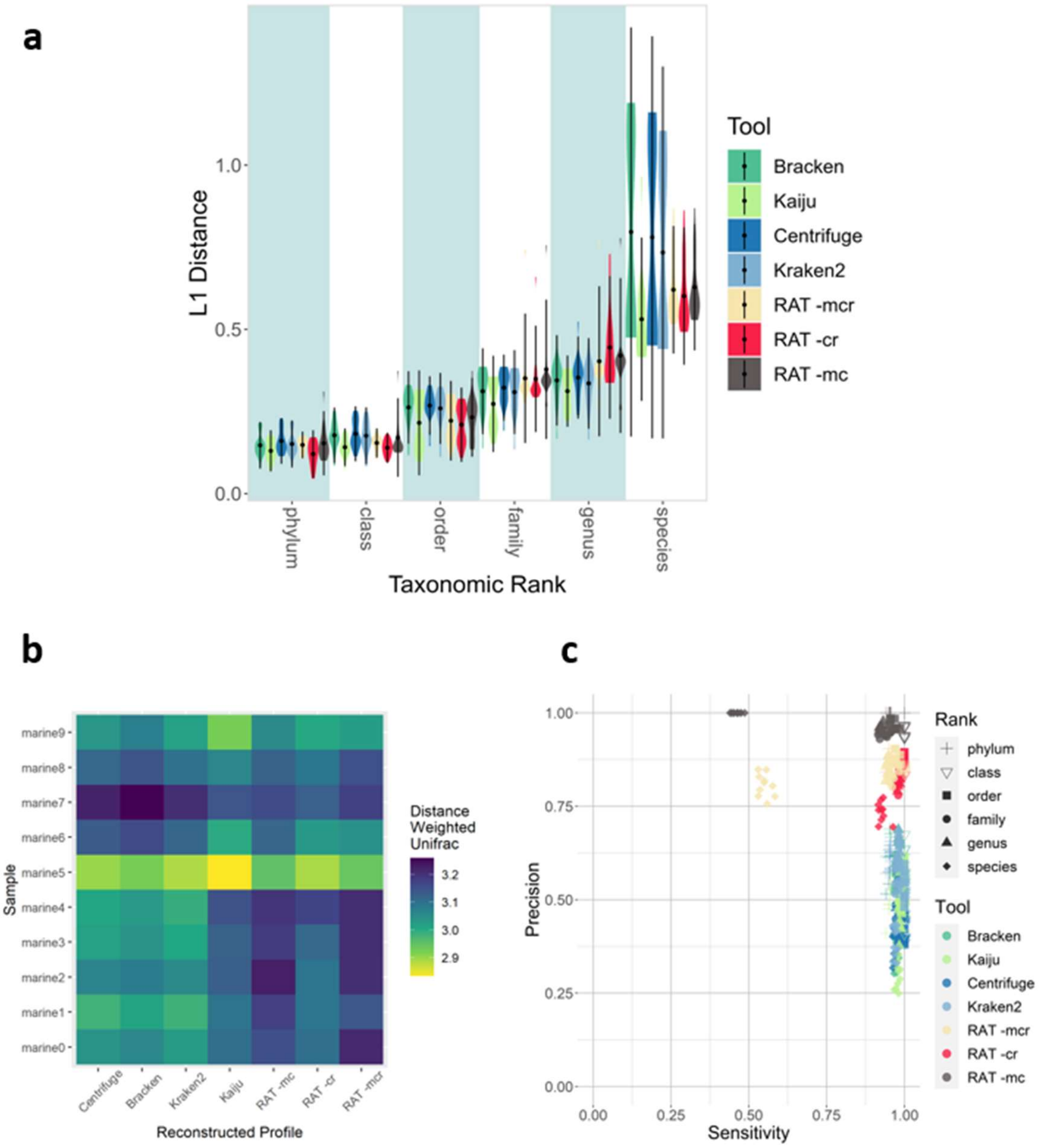

Supplementary Fig. 4 | Similarities between true profiles and profiles reconstructed by different tools of the CAMI2 marine dataset. We only counted taxa as detected if their relative abundance was at least 0.001% (a minimum of 4 reads). a, L1 values between profiles reconstructed by RAT/other tools and the true profiles (n=10 samples, error bars=standard deviation). An L1 value of 0 means that two profiles are identical (thus lower is better). The blue background is to facilitate recognizing which violin belongs to which rank. b, Heatmap of weighted UniFrac distances between reconstructed and true profiles (a shorter distance is better). c, Sensitivity vs. precision of the different tools. Different shapes signify the sensitivity/precision on different taxonomic ranks, different colours indicate tools (high precision + sensitivity is better). Source data are provided as a Source Data file.

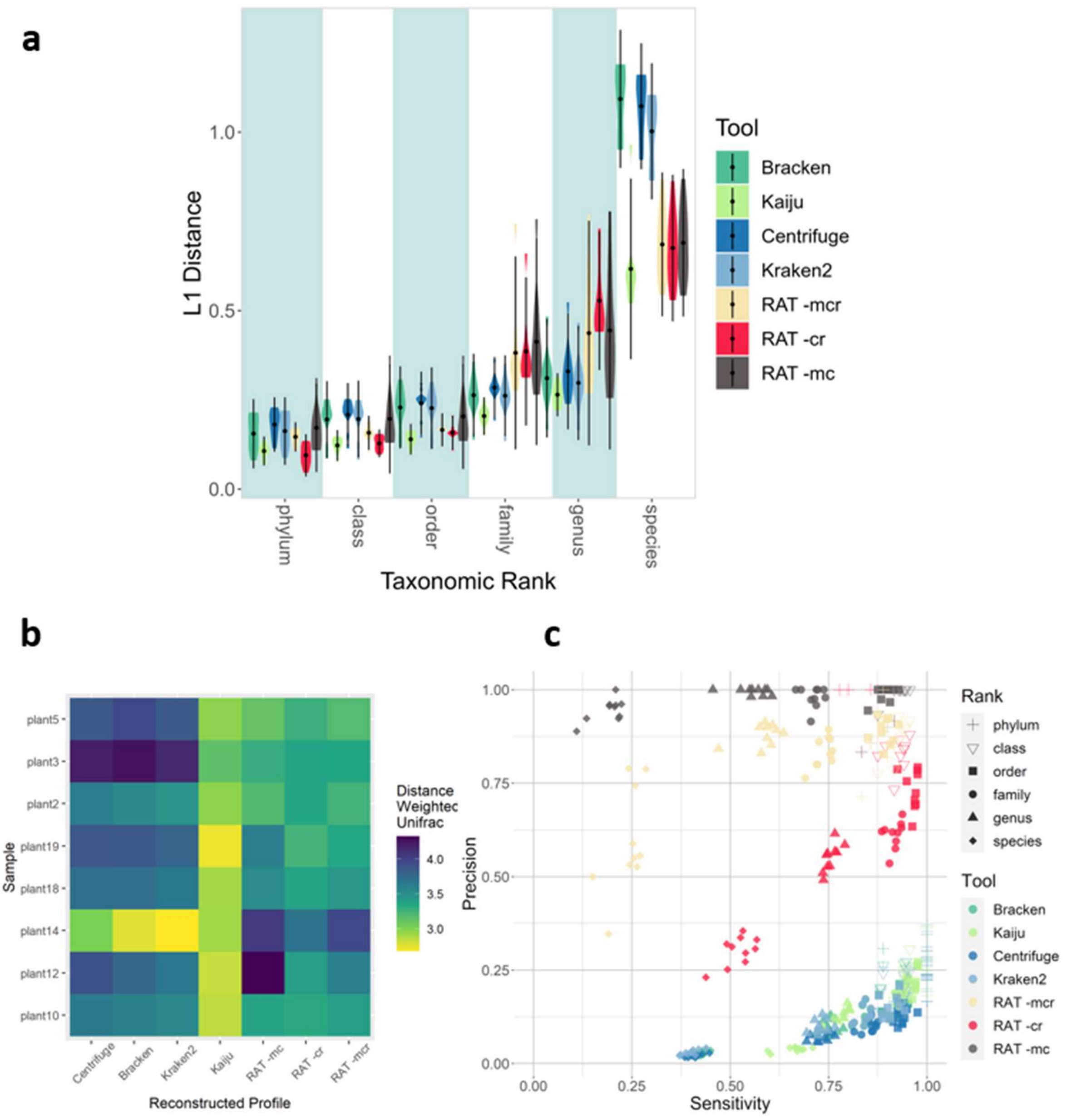

Supplementary Fig. 5 | Similarities between true profiles and profiles reconstructed by different tools of the CAMI2 rhizosphere dataset. We only counted taxa as detected if their relative abundance was at least 0.001% (a minimum of 4 reads). a, L1 values between profiles reconstructed by RAT/other tools and the true profiles (n=10 samples, error bars=standard deviation). An L1 value of 0 means that two profiles are identical (thus lower is better). The blue background is to facilitate recognizing which violin belongs to which rank. b, Heatmap of weighted UniFrac distances between reconstructed and true profiles (a shorter distance is better). c, Sensitivity vs. precision of the different tools. Different shapes signify the sensitivity/precision on different taxonomic ranks, different colours indicate tools (high precision + sensitivity is better). Source data are provided as a Source Data file.

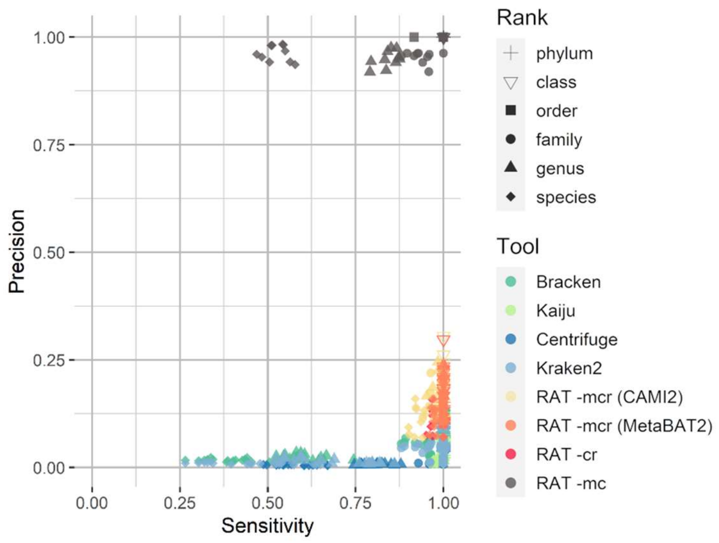

Supplementary Fig. 6 | Sensitivity versus precision of the different tools without an abundance cut-off to include taxa. Different shapes signify the sensitivity/precision on different ranks, different colours indicate tools (high precision + sensitivity is better). Source data are provided as a Source Data file.

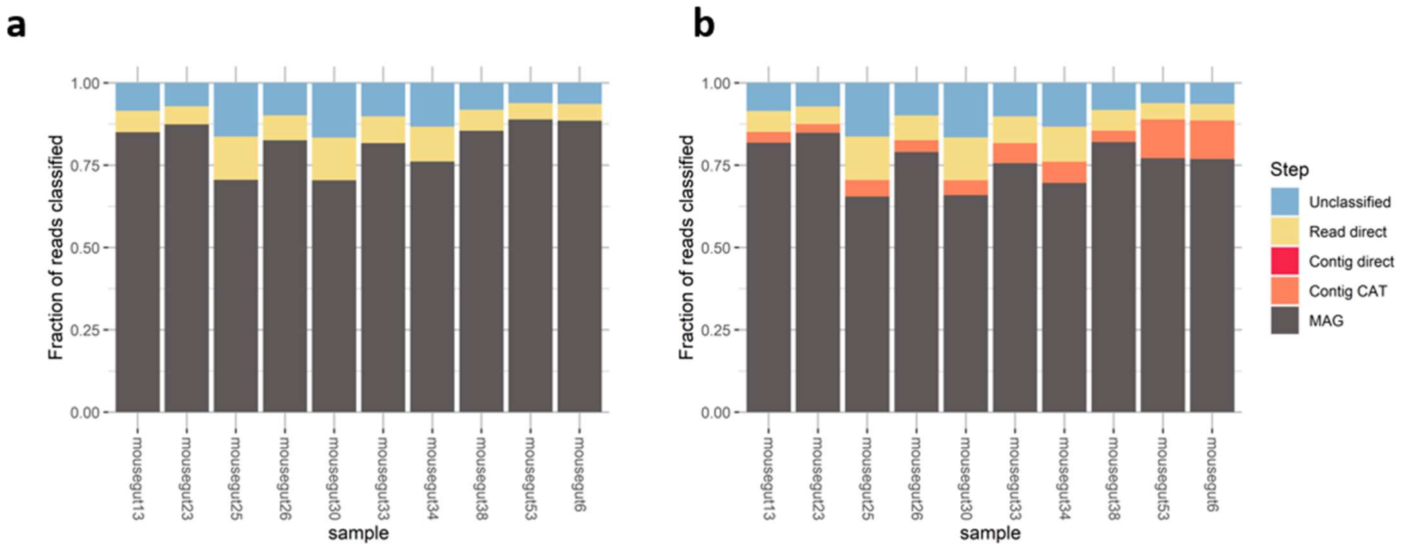

Supplementary Fig. 7 | Fraction of read annotations in the simulated CAMI dataset and the taxonomic signal (i.e. MAG, contig, direct read/contig mapping) they originate from. 'bin' refers to BAT annotation of a MAG, 'contig' refers to CAT annotation of a contig, 'contig_dm' and 'read_dm' refer to DIAMOND blastx direct annotations of contigs/reads. a, Read annotations per taxonomic signal using CAMI genomes as MAG input. b, Read annotations per taxonomic signal using MAGs binned by MetaBAT 2. Source data are provided as a Source Data file.

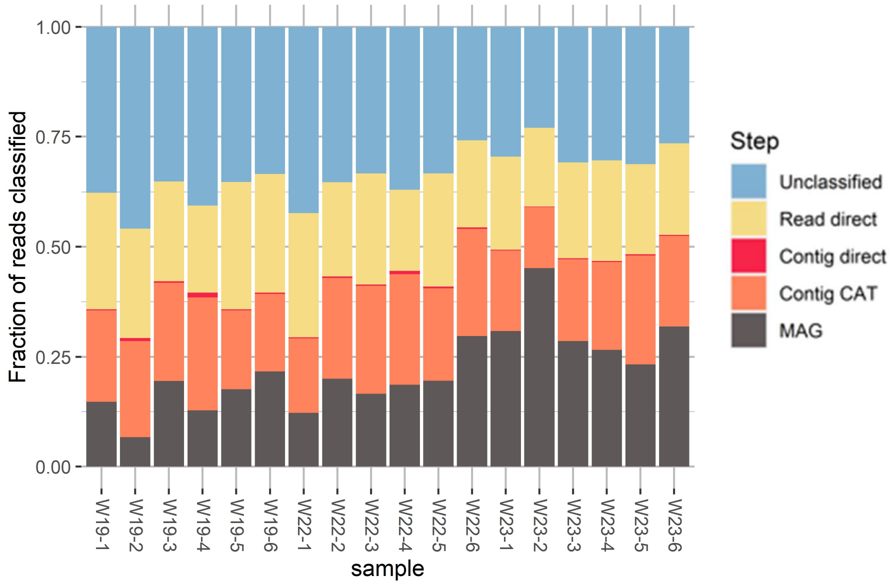

Supplementary Fig. 8 | Fraction of read annotations in the biological groundwater dataset and the taxonomic signal they originate from using the nr database. 'MAG' refers to BAT annotation of a MAG, 'Contig CAT' refers to CAT annotation of a contig, 'Contig direct' and 'Read direct' refer to DIAMOND blastx direct annotations of contigs/reads. Source data are provided as a Source Data file.

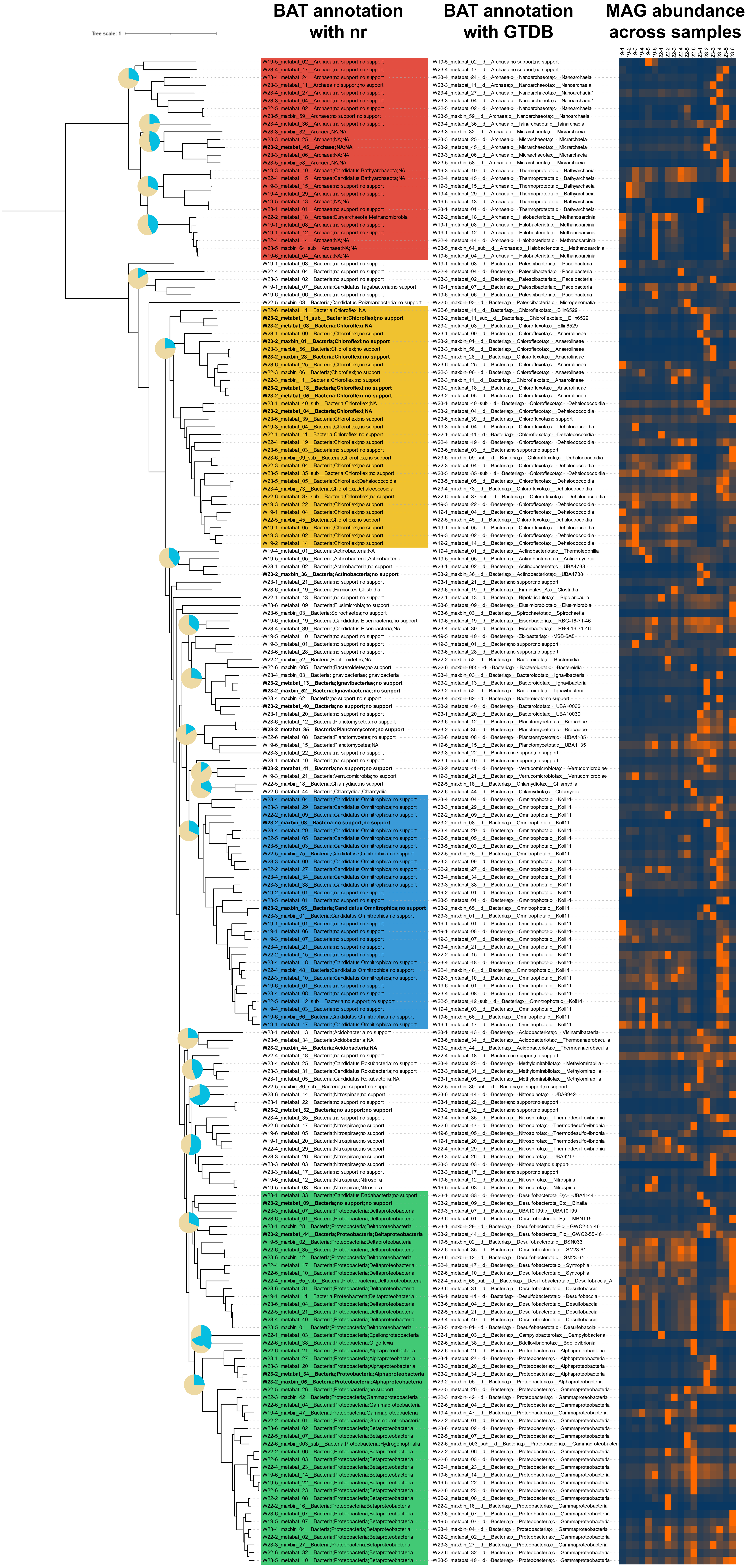

Tree scale: 1
BAT annotation with nr
BAT annotation with GTDB
MAG abundance across samples

Supplementary Fig. 9 | Phylogenetic tree of dereplicated MAGs from groundwater samples based on a concatenation of 43 universal marker genes. Colors behind the leaf labels highlight the members of large clusters of microbes (red - *Archaea*, yellow - *Chloroflexi*, blue - *Candidatus Omnitrophica*, green - *Proteobacteria*). Pie charts indicate fraction of reads per phylum that are mapped to MAGs (in blue) or to contigs or reads (in beige). MAG annotations are based on BAT with nr (left) and GTDB (right) as reference database. Taxonomic lineages are displayed down to taxonomic rank class. The heatmap on the right indicates normalised relative abundance of the MAG in each sample, with its highest relative abundance displayed in orange. Abundance is based on reads that map to any MAG in the dRep cluster. Source data are provided as a Source Data file.

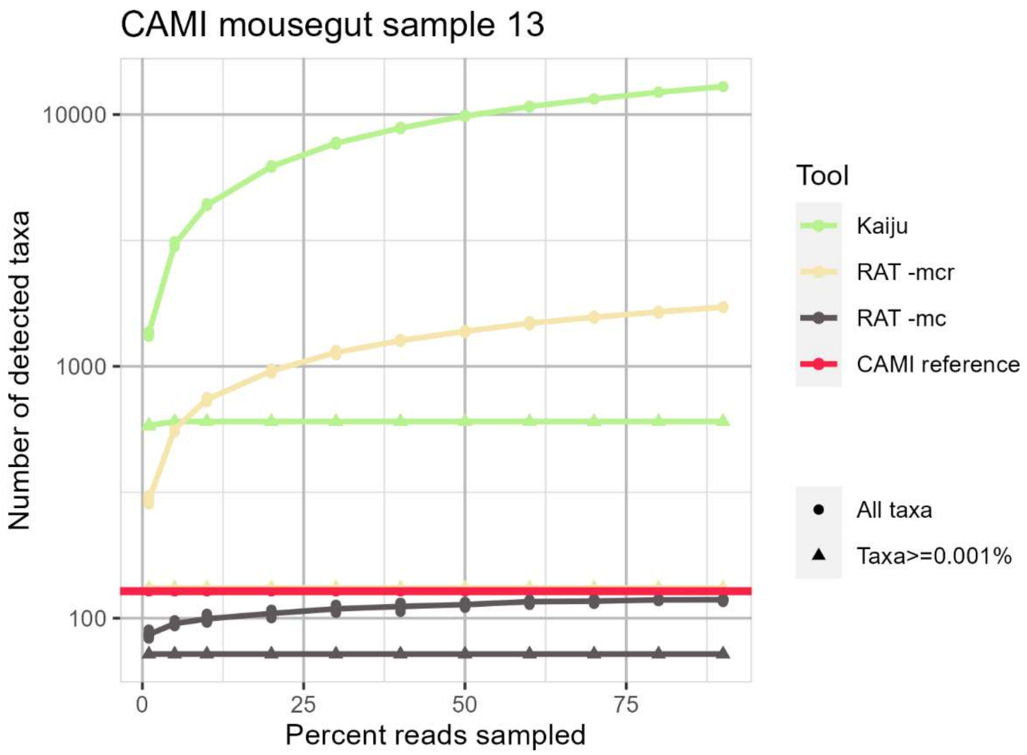

Supplementary Fig. 10 | Rarefaction curve of number of taxa detected in one of the simulated CAMI datasets by RAT -mc, RAT -mcr, and Kaiju (100 iterations). 'CAMI reference' refers to the actual number of taxa present in the sample. Triangles indicate the number of taxa detected in profiles when a minimum abundance is required to consider an organism as detected. Circles indicate the number of taxa detected without a cut-off. Source data are provided as a Source Data file.

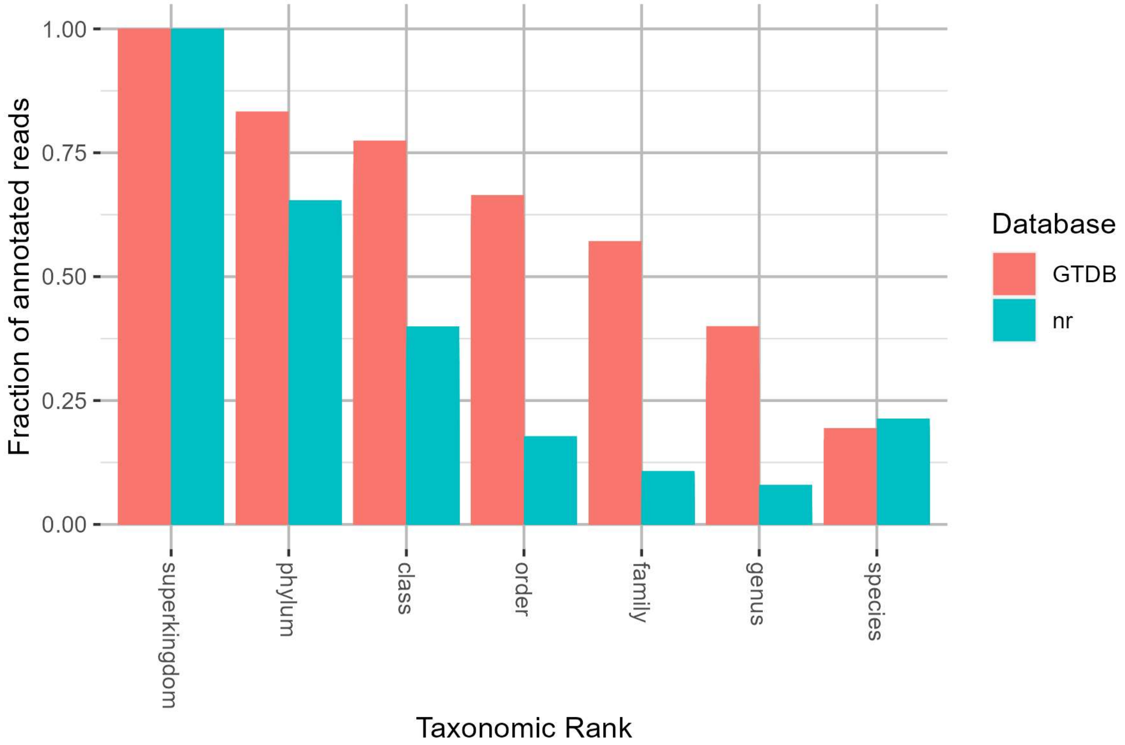

Supplementary Fig. 11 | Fraction of annotated reads across 18 biological samples depending on reference database. Bars indicate fraction of reads with available annotation, color of bars indicates the database. Source data are provided as a Source Data file.

Supplementary Tables

Supplementary Table 1 | General data on the samples of the CAMI2 challenge datasets.

environment	sample	number of reads	reads in bins	no species annotation	no genus annotation	median centrifuge hit length (~similarity to database)	known species
mouse gut	6	33098456	29310425	0	0	0	212
mouse gut	13	33184772	28225055	0	0	24	112
mouse gut	23	33204412	29033861	0	0	24	102
mouse gut	25	33255620	23466757	0	0	24	201
mouse gut	26	33216952	27433795	0	0	28	101
mouse gut	30	33283734	23444271	0	0	24	207
mouse gut	33	33261724	27189805	0	0	23	204
mouse gut	34	33246814	25312259	0	0	30	225
mouse gut	38	33185866	28365974	0	0	23	97
mouse gut	53	33080526	29411170	0	0	0	154
marine	0	33294790	30110766	4408072	199372	195	256
marine	1	33301262	29805514	3611876	96336	197	300
marine	2	33298832	29915265	4270212	261904	196	274
marine	3	33286888	29749450	4600016	223730	194	381
marine	4	33297894	29607554	6062018	200046	192	277
marine	5	33298452	29829072	4902650	63336	195	331
marine	6	33293400	29744097	4457860	100360	195	326
marine	7	33291302	29915566	4880020	150486	194	282
marine	8	33304294	30108029	4557290	283624	196	345
marine	9	33299844	29756592	5849204	254960	194	271
rhizosphere	2	33243296	32741216	8243786	2782466	68	78
rhizosphere	3	33249018	32646595	6058998	5653724	48	127
rhizosphere	5	33269696	32209513	5263526	4277212	57	96
rhizosphere	10	33220936	30709965	3293412	1994396	69	117
rhizosphere	12	33271926	24136023	12096308	487454	47	94
rhizosphere	14	33292604	32128543	2684076	684408	49	118
rhizosphere	18	33246400	29934037	6320158	1396876	53	116
rhizosphere	19	33246223	29424412	6505704	655986	53	107

Supplementary Table 2 | Runtime and maximum RAM usage of assembly and binning on two samples from the CAMI2 challenge.

Sample	Mousegut6	Mousegut13
Runtime assembly with MetaSPAdes	2h 35min	2h 0min
Max RAM MetaSPAdes	32.8 GB	26.1 GB
Runtime binning with MetaBAT2	2min	2min
Max RAM MetaBAT2	406 MB	172 MB
Runtime CAT + BAT	24h 14min	24h 14min

Supplementary Table 3 | Number of reads sequenced in the groundwater samples.

Sample	Reads sequenced
W19-1	36,438,396
W19-2	35,289,790
W19-3	42,407,036
W19-4	42,875,403
W19-5	38,591,389
W19-6	39,998,587
W22-1	42,251,696
W22-2	40,904,059
W22-3	43,615,855
W22-4	41,004,335
W22-5	39,219,955
W22-6	58,902,006
W23-1	41,964,387
W23-2	40,896,174
W23-3	42,448,523
W23-4	41,463,768
W23-5	47,496,123
W23-6	46,181,232

Supplementary Table 4 | Fraction of reads with a superkingdom annotation by Kaiju and RAT in the groundwater samples.

Sample	RAT	Kaiju
W19-1	0.654212	0.646401
W19-2	0.562158	0.510839
W19-3	0.681671	0.642909
W19-4	0.611402	0.494389
W19-5	0.673378	0.666271
W19-6	0.693093	0.677197
W22-1	0.598599	0.614838
W22-2	0.67577	0.643249
W22-3	0.695687	0.668228
W22-4	0.657298	0.571904
W22-5	0.690264	0.646235
W22-6	0.77066	0.696525
W23-1	0.738719	0.676679
W23-2	0.791681	0.665519
W23-3	0.715761	0.631563
W23-4	0.719839	0.668849
W23-5	0.716869	0.667231
W23-6	0.760786	0.698729

Supplementary Table 5 | MAG annotations with nr vs. GTDB for groundwater data. The columns named 'rank MAGs' refer to the number of MAGs at that rank that have an annotation with the given database. The columns named 'rank all' refer to the fraction of reads that get an annotation at that rank using RAT's integrated profiling approach with the given database.

sample	DB	MAGs	phylum MAGs	class MAGs	order MAGs	family MAGs	genus MAGs	species MAGs	phylum all	class all	order all	family all	genus all	species all
W19-1	GTDB	28	25	24	17	12	8	0	0.734	0.680	0.540	0.421	0.287	0.143
W19-1	nr	28	14	4	1	0	0	0	0.512	0.249	0.057	0.037	0.025	0.170
W19-2	GTDB	9	9	9	8	6	3	2	0.649	0.599	0.493	0.400	0.287	0.194
W19-2	nr	9	2	0	0	0	0	0	0.483	0.213	0.089	0.059	0.044	0.214
W19-3	GTDB	23	21	19	10	8	6	0	0.709	0.652	0.477	0.351	0.243	0.140
W19-3	nr	23	16	5	1	1	1	4	0.587	0.271	0.057	0.039	0.028	0.164
W19-4	GTDB	17	16	14	14	9	9	1	0.635	0.578	0.500	0.393	0.298	0.173
W19-4	nr	17	12	3	1	1	0	3	0.467	0.197	0.072	0.052	0.035	0.198
W19-5	GTDB	21	20	20	17	12	7	2	0.768	0.716	0.603	0.437	0.268	0.149
W19-5	nr	21	14	7	2	1	1	4	0.550	0.259	0.072	0.047	0.028	0.160
W19-6	GTDB	26	24	23	17	15	6	0	0.787	0.730	0.615	0.533	0.327	0.144
W19-6	nr	26	14	6	2	1	1	2	0.504	0.229	0.073	0.053	0.037	0.169
W22-1	GTDB	13	13	12	11	8	7	2	0.738	0.687	0.601	0.497	0.400	0.170
W22-1	nr	13	9	6	3	0	1	0	0.572	0.355	0.078	0.052	0.037	0.177
W22-2	GTDB	17	17	17	17	14	7	1	0.768	0.725	0.625	0.500	0.292	0.150
W22-2	nr	17	15	10	6	3	2	2	0.624	0.330	0.172	0.104	0.035	0.160
W22-3	GTDB	22	20	19	16	10	5	0	0.750	0.706	0.589	0.451	0.263	0.136
W22-3	nr	22	16	4	1	1	1	1	0.617	0.338	0.132	0.108	0.076	0.181
W22-4	GTDB	29	27	24	20	16	9	3	0.737	0.681	0.551	0.421	0.258	0.166
W22-4	nr	29	20	7	1	0	1	3	0.543	0.294	0.111	0.082	0.080	0.182
W22-5	GTDB	31	29	28	27	20	8	0	0.759	0.710	0.612	0.498	0.279	0.146
W22-5	nr	31	21	7	1	0	0	2	0.571	0.250	0.098	0.059	0.039	0.165
W22-6	GTDB	38	38	35	30	27	11	1	0.828	0.775	0.665	0.572	0.324	0.138
W22-6	nr	38	37	19	9	2	1	3	0.604	0.368	0.178	0.100	0.071	0.142
W23-1	GTDB	37	33	30	26	19	4	2	0.784	0.729	0.643	0.552	0.245	0.131
W23-1	nr	37	19	4	0	0	0	5	0.548	0.127	0.037	0.024	0.019	0.145
W23-2	GTDB	46	44	43	39	28	6	2	0.833	0.771	0.606	0.469	0.224	0.088
W23-2	nr	46	29	4	0	0	0	8	0.575	0.142	0.034	0.024	0.017	0.114
W23-3	GTDB	45	41	38	30	23	8	3	0.775	0.640	0.544	0.433	0.224	0.133
W23-3	nr	45	18	6	1	0	0	8	0.462	0.180	0.044	0.029	0.022	0.143
W23-4	GTDB	42	38	35	29	24	10	4	0.782	0.724	0.606	0.510	0.272	0.168
W23-4	nr	42	22	8	2	0	1	5	0.524	0.203	0.041	0.022	0.022	0.152
W23-5	GTDB	37	36	33	27	25	9	3	0.786	0.730	0.574	0.474	0.252	0.158
W23-5	nr	37	25	11	2	1	2	4	0.482	0.208	0.038	0.025	0.024	0.160
W23-6	GTDB	33	29	27	26	21	12	2	0.747	0.683	0.620	0.492	0.301	0.126
W23-6	nr	33	24	11	1	0	0	1	0.654	0.400	0.053	0.033	0.022	0.123
