# Supplementary material for: Integrating taxonomic signals from MAGs and contigs improves read annotation and taxonomic profiling of metagenomes
Source: Nat Commun. 2024 Apr 20;15:3373. doi: 10.1038/s41467-024-47155-1 (PMC11032395; doi:10.1038/s41467-024-47155-1)
Supplement: Supplementary file 3 — Reporting Summary [file 41467_2024_47155_MOESM3_ESM.pdf]

nature portfolio

Corresponding author(s): F. A. Bastiaan von Meijenfeldt

Last updated by author(s): Mar 11, 2024

Reporting Summary

Nature Portfolio wishes to improve the reproducibility of the work that we publish. This form provides structure for consistency and transparency in reporting. For further information on Nature Portfolio policies, see our Editorial Policies and the Editorial Policy Checklist.

Statistics

For all statistical analyses, confirm that the following items are present in the figure legend, table legend, main text, or Methods section.

n/a	Confirmed	
☐	☒	The exact sample size (*n*) for each experimental group/condition, given as a discrete number and unit of measurement
☐	☒	A statement on whether measurements were taken from distinct samples or whether the same sample was measured repeatedly
☒	☐	The statistical test(s) used AND whether they are one- or two-sided<br>*Only common tests should be described solely by name; describe more complex techniques in the Methods section.*
☐	☒	A description of all covariates tested
☐	☒	A description of any assumptions or corrections, such as tests of normality and adjustment for multiple comparisons
☐	☒	A full description of the statistical parameters including central tendency (e.g. means) or other basic estimates (e.g. regression coefficient) AND variation (e.g. standard deviation) or associated estimates of uncertainty (e.g. confidence intervals)
☒	☐	For null hypothesis testing, the test statistic (e.g. *F*, *t*, *r*) with confidence intervals, effect sizes, degrees of freedom and *P* value noted<br>*Give P values as exact values whenever suitable.*
☒	☐	For Bayesian analysis, information on the choice of priors and Markov chain Monte Carlo settings
☒	☐	For hierarchical and complex designs, identification of the appropriate level for tests and full reporting of outcomes
☒	☐	Estimates of effect sizes (e.g. Cohen's *d*, Pearson's *r*), indicating how they were calculated

Our web collection on statistics for biologists contains articles on many of the points above.

Software and code

Policy information about availability of computer code

Data collection	No software was used for data collection.
Data analysis	Data was analyzed with Read Annotation Tool available at https://github.com/MGXlab/CAT_pack. Within the RAT pipeline, we also run Prodigal (https://github.com/hyattpd/Prodigal), DIAMOND (https://github.com/bbuchfink/diamond), bwa mem (https://github.com/lh3/bwa), and samtools (https://www.htslib.org/). We further used the following tools to analyze data: Centrifuge (https://ccb.jhu.edu/software/centrifuge/), Kaiju (https://bioinformatics-centre.github.io/kaiju/), Kraken2 (https://ccb.jhu.edu/software/kraken2/). We binned data using MetaBAT2 (https://bitbucket.org/berkeleylab/metabat/src/master/). On the biological samples, we used the ATLAS pipeline (https://github.com/metagenome-atlas/atlas).

For manuscripts utilizing custom algorithms or software that are central to the research but not yet described in published literature, software must be made available to editors and reviewers. We strongly encourage code deposition in a community repository (e.g. GitHub). See the Nature Portfolio guidelines for submitting code & software for further information.

Data

Policy information about availability of data

All manuscripts must include a data availability statement. This statement should provide the following information, where applicable:

- Accession codes, unique identifiers, or web links for publicly available datasets
- A description of any restrictions on data availability
- For clinical datasets or third party data, please ensure that the statement adheres to our policy

RAT is available on GitHub at https://github.com/MGXlab/CAT_pack. The scripts used in the downstream analyses are available on GitHub at https://github.com/thauptfeld/RAT_paper. The raw sequencing reads and assemblies used for the biological analyses are available at SRA under BioProject ID PRJNA947390. Data from the CAMI2 challenge is available at https://data.cami-challenge.org/participate.

Research involving human participants, their data, or biological material

Policy information about studies with human participants or human data. See also policy information about sex, gender (identity/presentation), and sexual orientation and race, ethnicity and racism.

Reporting on sex and gender	N/A
Reporting on race, ethnicity, or other socially relevant groupings	N/A
Population characteristics	N/A
Recruitment	N/A
Ethics oversight	N/A

Note that full information on the approval of the study protocol must also be provided in the manuscript.

Field-specific reporting

Please select the one below that is the best fit for your research. If you are not sure, read the appropriate sections before making your selection.

☐ Life sciences ☐ Behavioural & social sciences ☒ Ecological, evolutionary & environmental sciences

For a reference copy of the document with all sections, see nature.com/documents/nr-reporting-summary-flat.pdf

Ecological, evolutionary & environmental sciences study design

All studies must disclose on these points even when the disclosure is negative.

Study description	We developed a bioinformatic tool to estimate taxonomic composition from metagenomes by integrating the assembly and MAGs into the taxonomic annotation of each read. To benchmark our tool, we tested our method on 28 samples of simulated metagenomes in comparison with different state-of-the-art taxonomic profilers. We also tested our tool on 18 groundwater metagenomes from 3 groundwater monitoring wells in the Netherlands.
Research sample	We used 10 samples from the toy mouse gut dataset from second CAMI challenge, because it was the earliest available dataset. We also included 10 marine samples and 8 (originally 10) samples from the rhizosphere dataset, also of the second CAMI challenge.
Sampling strategy	We randomly sampled 10 samples per dataset using the R function random.sample(). For the biological dataset we included samples of all available depths per well.
Data collection	We downloaded the simulated metagenomes from https://data.cami-challenge.org/participate. We collected 6-10 L of groundwater per sample and filtered it through a 0.2µm filter to catch cellular organisms.
Timing and spatial scale	We sampled the biological datasets on 2 different days in June 2018.
Data exclusions	Of the rhizosphere samples 2 were excluded, because >1,000 of the supposed reference taxids were not present in the database provided by the CAMI challenge.
Reproducibility	As our study focuses on the bioinformatic method and all the sequence data as well as the code is public on a database/a repository, anyone can repeat the study at any given time.
Randomization	Not applicable
Blinding	As we are merely using scripts to compare annotations to the reference profile, blinding is not applicable in our study.

Did the study involve field work? ☒ Yes ☐ No

Field work, collection and transport

Field conditions	Cloudy, no rain, approximately 20 degrees. Aquifer temperature 10-12 degrees.
Location	Northeast Netherlands (exact location subject to NDA)
Access & import/export	Monitoring well next to a village street, import/export not applicable
Disturbance	No disturbance, as monitoring well is already present and regularly used the same way by Vitens (drinking water provider for the Netherlands)

Reporting for specific materials, systems and methods

We require information from authors about some types of materials, experimental systems and methods used in many studies. Here, indicate whether each material, system or method listed is relevant to your study. If you are not sure if a list item applies to your research, read the appropriate section before selecting a response.

Materials & experimental systems

n/a	Involved in the study
☒	☐ Antibodies
☒	☐ Eukaryotic cell lines
☒	☐ Palaeontology and archaeology
☒	☐ Animals and other organisms
☒	☐ Clinical data
☒	☐ Dual use research of concern
☒	☐ Plants

Methods

n/a	Involved in the study
☒	☐ ChIP-seq
☒	☐ Flow cytometry
☒	☐ MRI-based neuroimaging

Plants

Seed stocks	N/A
Novel plant genotypes	N/A
Authentication	N/A
